# Safety of Tyrosine Kinase Inhibitors in Patients With Differentiated Thyroid Cancer: Real-World Use of Lenvatinib and Sorafenib in Korea

**DOI:** 10.3389/fendo.2019.00384

**Published:** 2019-06-12

**Authors:** Soo Young Kim, Seok-Mo Kim, Hojin Chang, Bup-Woo Kim, Yong Sang Lee, Hang-Seok Chang, Cheong Soo Park

**Affiliations:** Department of Surgery, Thyroid Cancer Center, Gangnam Severance Hospital, Yonsei University College of Medicine, Seoul, South Korea

**Keywords:** adverse effects, chart review, differentiated thyroid cancer, lenvatinib, refractory thyroid cancer, safety, sorafenib, tyrosine kinase inhibitors

## Abstract

**Background:** Thyroid cancer has become the most common cancer in Korea. Generally, thyroid cancer patients have a good prognosis; however, 15–20% of patients experience recurrence or distant metastasis or are refractory to standard treatment. We assessed the safety of sorafenib and lenvatinib in patients with advanced or metastatic radioactive iodine-refractory differentiated thyroid cancers (DTC) consecutively treated at a tertiary center in South Korea.

**Methods:** We retrospectively reviewed the charts of all consecutive patients with DTC treated during ≥6 months with lenvatinib (February 2016–April 2018) and sorafenib (January 2014–April 2018) at Gangnam Severance Hospital. Patients were treated according to the prescribing information of each drug and were followed up for 2 months. We evaluated the adverse events (AEs) reported with each drug.

**Results:** A total of 71 medical records (lenvatinib, *n* = 23; sorafenib, *n* = 48) were reviewed. The most common histological types were papillary thyroid cancer (69.0%) and follicular thyroid cancer (22.5%). All patients (*n* = 23) started lenvatinib at a dose of 20 mg; 41.7% of sorafenib-treated patients received an initial dose of 800 mg daily. Four (17.4%) lenvatinib-treated patients and 26 (54.2%) sorafenib-treated patients required treatment discontinuation. The most common AEs of any grade in the lenvatinib group were diarrhea (82.6%), hypertension (78.3%), hand-foot skin reaction (56.5%), weight loss (52.2%), proteinuria (47.8%), and anorexia (43.5%). In the sorafenib group, these were hand-foot skin reaction (87.5%), diarrhea (62.5%), anorexia (60.4%), alopecia (56.3%), mucositis (52.1%), weight loss and generalized weakness (each, 50%), and hypertension (43.8%). The incidence of hand-foot skin reaction, alopecia, and rash of any grade was significantly lower (*P* = 0.003, *P* = 0.017, and *P* = 0.017) in patients treated with lenvatinib compared with those treated with sorafenib. The incidence of hypertension, QT prolongation, and proteinuria of any grade was significantly higher (*P* = 0.006, *P* = 0.038, and *P* < 0.001) in patients treated with lenvatinib compared with those treated with sorafenib. Seven deaths occurred, which were attributed to disease progression.

**Conclusions:** No new safety concerns were identified for either drug. Most AEs were managed with dose modification and medical therapy. AEs such as hypertension and proteinuria warrant close monitoring.

## Introduction

In recent decades, the incidence of thyroid cancer has increased considerably both worldwide (1) and in Korea (2). In Korea, the incidence of thyroid cancer increased by 22.3% per year in both sexes, and thyroid cancer is the most common cancer since 2009 (2). The most common histological subtypes of thyroid cancer are papillary thyroid cancer (PTC), follicular thyroid cancer (FTC), poorly differentiated thyroid cancer, and anaplastic thyroid cancer. PTC and FTC are collectively classified as differentiated thyroid cancers (DTC).

Although most thyroid cancer patients have good overall survival, about 15–20% of patients experience recurrence or distant metastasis (3–5), are refractory to standard treatment (6, 7), or experience considerable treatment-related toxicities. There are limited therapeutic options for such patients with advanced thyroid cancer and poor prognosis (7); thus, safe and tolerable treatment options are needed.

The molecular signals involved in the pathogenesis of thyroid cancer are the RAS and BRAF/MEK/ERK signaling pathways; ligand-independent RET/PTC receptor tyrosine kinase activation; and pathways involving vascular endothelial growth factor (VEGF), platelet-derived growth factor (PDGF), and their receptors (8, 9). Lenvatinib is a multitargeted tyrosine kinase inhibitor (TKI) that targets VEGF receptors 1–3, fibroblast growth factor receptors (FGFR) 1–4, PDGF receptor a, RET, and KIT (10, 11). Lenvatinib was shown to be efficacious in patients with advanced, progressive DTC refractory to radioactive iodine in the randomized phase 3 SELECT trial (12), with a median progression-free survival (PFS) of 18.3 months compared with 3.6 months in the placebo group. Based on the results of the SELECT trial, lenvatinib was approved to treat patients with advanced and progressive DTC refractory to radioactive iodine (RAI). In Korea, lenvatinib was approved and launched into the market in February 2016.

Sorafenib is a multitargeted TKI that blocks the activity of Raf serine/threonine kinase isoforms, VEGF receptor-2 and−3, PDGF receptor β, c-KIT, FMS-like tyrosine kinase (FLT)-3, and RET, resulting in inhibition of tumor angiogenesis and cell proliferation (13–15). In the phase 3 DECISION trial, sorafenib treatment significantly improved PFS compared with placebo (*P* < 0.0001; median 10.8 vs. 5.8 months, respectively) (16). Sorafenib was approved and launched into the market in Korea in January 2014.

As lenvatinib was recently approved for the treatment of DTC, there is a lack of efficacy and safety data. The mechanism of action of lenvatinib differs from that of sorafenib, and thus, it may have a different safety profile. The lack of real-world clinical data and the presumed differences in safety profiles may preclude clinicians from using lenvatinib in their routine clinical practice. The present study aimed to evaluate the safety of lenvatinib and sorafenib in consecutive patients with advanced or metastatic RAI-refractory DTC treated at a tertiary center in a real-world clinical practice setting in Korea.

## Methods

### Study Design, Setting, and Treatment

This was a retrospective medical chart review conducted in Gangnam Severance Hospital, Seoul, South Korea. Charts of patients treated with lenvatinib from February 2016 to April 2018 and those of patients treated with sorafenib from January 2014 to April 2018 were retrospectively reviewed. Sorafenib was used to treat all patients prior to lenvatinib becoming available in hospitals in February 2016. This study focused only on the adverse events (AEs) reported with each treatment.

Treatments were prescribed by the treating physician according to the prescribing information for each drug (LENVIMA^®^ [lenvatinib] capsules, for oral use. Highlights of prescribing information 2018^1^; NEXAVAR^®^ [sorafenib] tablets, oral. Highlights of prescribing information 2013^2^), based on their judgment and local practices. For patients who underwent treatment for a minimum of 6 months, the follow-up interval was 2 months, and all patients had three or more follow-up visits.

The study was carried out in accordance with the principles laid out in the World Medical Association's Declaration of Helsinki, Good Clinical Practice, and associated Korean regulations. This study was approved by the Institutional Review Board of Gangnam Severance Hospital (approval number 2017-0116-004). As data were obtained retrospectively, patient identities remained anonymous, and informed consent is not mandatory for retrospective studies in Korea, the institutional review board waived the need for informed consent.

### Patients

In this study, we included all consecutive patients with DTC who were treated with lenvatinib or sorafenib for more than 6 months at our center during the designated chart review period.

### TKI Use

TKI use was assessed in terms of initial and final doses administered, as well as dose reductions, drug interruptions, or discontinuations because of AEs.

### Safety

To assess the safety of the treatment, adverse event data were obtained retrospectively from hospital records and were classified according to the National Cancer Institute Common Terminology Criteria for Adverse Events v4.0. Safety assessments also included physical examination, electrocardiogram, and laboratory testing (including blood cell count, liver and renal function, electrolyte levels, and proteinuria), during follow-up visits.

### Statistical Analysis

The sample size was not calculated or preplanned as this study aimed to evaluate the records of all consecutive patients with DTC treated at our center during the study period. Descriptive analyses were performed. Quantitative data are expressed as absolute numbers and median or mean. Qualitative data are expressed as a percentage of the entire population (n [%]). Data in the two treatment groups were compared using the Student's *t*-test, the chi-square test, and Fisher's exact test, as appropriate. The statistical software used to perform the statistical analysis in this study was SPSS version 22 (SPSS Inc., Chicago, IL, USA).

## Results

### Patients

In total, 71 medical records of patients with advanced or metastatic DTC consecutively treated with lenvatinib (*n* = 23) or sorafenib (*n* = 48) at our center were analyzed. The main patient demographic and clinical characteristics are shown in Table 1. The median age of all patients at the start of treatment was 61.7 (32.6–79.0) years, which was similar among those treated with lenvatinib and sorafenib; 56.3% of patients were 60 years of age or older. Of the patients with DTC in the lenvatinib- and sorafenib-treated groups, 39.1 and 41.7% of patients were men. Distant metastases were observed in 88.7% of patients, and locally advanced DTC was identified in 11.3% of patients. The most common histological types were PTC (69.0%), followed by FTC (22.5%), and poorly differentiated thyroid cancer (7.1%). All patients had received previous RAI, and the median cumulative RAI was 665 mCi (30–4000) among those treated with lenvatinib and 730 mCi (30–1580) among those treated with sorafenib. Systemic therapy had been previously administered to 10 (43.5%) patients treated with lenvatinib; none of those treated with sorafenib had received prior systemic therapy.

**Table 1:** Patient demographic and clinical characteristics.

**Patients characteristics**	**Lenvatinib (*N* = 23)**	**Sorafenib (*N* = 48)**	**Total (*N* = 71)**	***P*-value**
**Age at the Start of Treatment, Years**				
Median (range)	59.7 (38.9–74.4)	62.0 (32.6–79.0)	61.7 (32.6–79.0)	0.961
≥60 years	11 (47.8)	29 (60.4)	40 (56.3)	0.317
Sex				0.839
Male	9 (39.1)	20 (41.7)	29 (40.8)	
Female	14 (60.9)	28 (58.3)	42 (59.2)	
Metastases				0.743
Locally advanced	3 (13.0)	5 (10.4)	8 (11.3)	
Distant	20 (87.0)	43 (89.6)	63 (88.7)	
ECOG performance status				0.971
0	7 (30.4)	16 (33.3)	23 (32.3)	
1	12 (52.2)	24 (50.0)	36 (50.7)	
2	4 (17.4)	8 (16.7)	12 (17.0)	
Histology				0.415
Papillary	14 (60.9)	35 (72.9)	49 (69.0)	
Follicular	7 (30.5)	9 (18.8)	16 (22.5)	
Hürthle cell	0	1 (2.1)	1 (1.4)	
Poorly differentiated	2 (8.6)	3 (6.3)	5 (7.1)	
**Most Common Metastatic Lesion Sites (Multiple)**				
Lung	18 (78.3)	41 (85.4)	59 (83.1)	0.451
Bone	9 (39.1)	16 (33.3)	25 (35.2)	0.632
Brain	0	3 (6.3)	3 (4.2)	0.221
Liver	1 (4.3)	2 (4.2)	3 (4.2)	0.972
Other (renal, mediastinal)	2 (8.7)	2 (4.2)	4 (5.6)	0.439
**Previous Treatment**				
Cumulative radioiodine activity, median (range), mCi	665 (30–4000)	730 (30–1580)	710 (30–4000)	0.966
Previous systemic therapy	10 (43.5)	0	10 (43.5)	0.018

Data in the table are presented as n (%), unless otherwise stated.

*ECOG, Eastern Cooperative Oncology Group*.

### TKI Use

The use of lenvatinib and sorafenib during the study period is shown in Table 2. All patients (*n* = 23) started lenvatinib at the dose of 20 mg. Among sorafenib-treated patients, 41.7% received an initial dose of 800 mg daily. At the end of the treatment, only 14.6% of patients were receiving the 800 mg daily dose; 41.7 and 43.8% of patients were receiving reduced sorafenib doses of ≤400 mg daily and 600 mg daily, respectively.

**Table 2:** Use of lenvatinib and sorafenib during the study period.

**Lenvatinib**	***N* = 23** ***n* (%)**
Initial lenvatinib dose (20 mg)	23 (100)
**Final Lenvatinib Dose**	
10 mg daily	7 (30.4)
14 mg daily	2 (8.7)
20 mg daily	14 (60.9)
Dose reduction for AEs	8 (34.8)
**Discontinuation of Lenvatinib**	
AE	1 (4.3)
Disease progression	0
Death	2 (8.7)
Financial issues	1 (4.3)
**Sorafenib**	***N*** **=** **48** ***n*** **(%)**
**Initial Sorafenib Dose**	
≤400 mg daily	12 (25.0)
600 mg daily	16 (33.3)
800 mg daily	20 (41.7)
**Final Sorafenib Dose**	
≤400 mg daily	20 (41.7)
600 mg daily	21 (43.8)
800 mg daily	7 (14.6)
Dose reduction for AEs	27 (56.3)
**Discontinuation of Sorafenib**	
AE	4 (8.3)
Disease progression	17 (35.4)
Death	5 (10.4)

*AE, adverse event*.

Eight (34.8%) lenvatinib-treated patients required dose reductions because of AE onset (some patients reported more than one AE: hand-foot skin reactions, *n* = 3; weight loss, *n* = 3; QT prolongation, *n* = 1; hypertension, *n* = 2; proteinuria, *n* = 3), while 27 (56.3%) sorafenib-treated patients required dose reduction because of AE onset (some patients reported more than one AE: hand-foot skin reactions, *n* = 24; general weakness, *n* = 7; headache, *n* = 2; hypertension, *n* = 2; weight loss, *n* = 7). Four (17.4%) lenvatinib-treated patients required treatment discontinuation; the reasons were death in two cases (8.7%), an AE in one case (4.3%), and financial issues in another case (4.3%). Twenty-six (54.2%) sorafenib-treated patients required treatment discontinuation; the reasons were disease progression in 17 cases (35.4%), death in five cases (10.4%), and AEs in four cases (8.3%; coronary artery occlusion, *n* = 1; weight loss, *n* = 1; uncontrolled hypertension, *n* = 1; and cerebrovascular disease, *n* = 1).

### Safety

The safety and tolerability findings are shown in Table 3, 4. In the lenvatinib group, the most common AEs of any grade with an incidence above 40% were diarrhea (82.6%), hypertension (78.3%), hand-foot skin reaction (56.5%), weight loss (52.2%), proteinuria (47.8%), and anorexia (43.5%). In the sorafenib group, the most common AEs of any grade with an incidence above 40% were hand-foot skin reaction (87.5%), diarrhea (62.5%), anorexia (60.4%), alopecia (56.3%), mucositis (52.1%), weight loss and generalized weakness (each, 50%), and hypertension (43.8%).

**Table 3:** Comparison of the incidence of adverse events of any grade in patients treated with lenvatinib vs. those treated with sorafenib.

**Adverse events *N* = 71**	**Lenvatinib *N* = 23** ***n* (%)**	**Sorafenib *N* = 48** ***n* (%)**	***P*-value**
Hand-foot skin reaction	13 (56.5)	42 (87.5)	0.003
Diarrhea	19 (82.6)	30 (62.5)	0.086
Alopecia	6 (26.1)	27 (56.3)	0.017
Rash	2 (8.7)	17 (35.4)	0.017
Mucositis	29 (39.1)	25 (52.1)	0.307
Hypertension	18 (78.3)	21 (43.8)	0.006
QT prolongation	2 (8.7)	0	0.038
Generalized weakness	9 (39.1)	24 (50)	0.390
Headache	1 (4.3)	5 (10.4)	0.390
Leucopenia	0	4 (8.3)	0.154
Anemia	1 (4.3)	4 (8.3)	0.539
Hepatitis	0	1 (2.1)	0.486
Anorexia	10 (43.5)	29 (60.4)	0.179
Weight loss	12 (52.2)	24 (50.0)	0.864
Cerebrovascular accident	1 (4.3)	2 (4.2)	0.972
Renal impairment	2 (8.7)	1 (2.1)	0.195
Proteinuria	11 (47.8)	0	<0.001

**Table 4:** Comparison of the incidence of Grade ≥3 adverse events in patients treated with lenvatinib vs. those treated with sorafenib.

**Adverse events *N* = 71**	**Lenvatinib *N* = 23** ***n* (%)**	**Sorafenib *N* = 48** ***n* (%)**	***P*-value**
Hand-foot skin reaction	7 (30.4)	32 (66.7)	0.004
Diarrhea	1 (4.3)	7 (14.6)	0.202
Rash	0	2 (4.2)	0.321
Mucositis	1 (4.3)	3 (6.3)	0.745
Hypertension	17 (73.9)	15 (31.3)	0.001
QT prolongation	2 (8.7)	0	0.038
Generalized weakness	1 (4.3)	6 (12.5)	0.281
Leucopenia	0	2 (4.2)	0.321
Hepatitis	0	1 (2.1)	0.486
Anorexia	1 (4.3)	10 (20.8)	0.072
Weight loss	0	4 (8.3)	0.154
Cerebrovascular accident	0	2 (4.2)	0.321
Renal impairment	0	0	
Proteinuria	2 (8.7)	0	0.038

When comparing the incidence of AEs of any grade between patients treated with lenvatinib and those treated with sorafenib, significantly fewer patients treated with lenvatinib had a hand-foot skin reaction (56.5 vs. 87.5%; *P* = 0.003), alopecia (26.1 vs. 56.3%; *P* = 0.017), and rash (8.7 vs. 35.4%; *P* = 0.017) (Table 3). Significantly more patients treated with lenvatinib had hypertension (78.3 vs. 43.8%; *P* = 0.006) (Table 3). Further, QT prolongation (2 [8.7%]) and proteinuria (11 [47.8%]) were only reported in patients treated with lenvatinib, and these between-group differences were significant (*P* = 0.038 and *P* < 0.001, respectively) (Table 3). In patients with QT prolongation, electrolyte levels and blood glucose were normal and, in some patients, no changes in thyroid function were observed.

When comparing the incidence of Grade ≥3 AEs between patients treated with lenvatinib and those treated with sorafenib, significantly less patients treated with lenvatinib presented a Grade ≥3 hand-foot skin reaction (30.4 vs. 66.7%; *P* = 0.004), but significantly more patients treated with lenvatinib presented Grade ≥3 hypertension (73.9 vs. 31.3%; *P* = 0.001) (Table 4). Grade ≥3 QT prolongation (2 [8.7%]) and proteinuria (2 [8.7%]) were only reported in patients treated with lenvatinib, and these between-group differences were significant (*P* = 0.038 and *P* <0.001, respectively) (Table 4). There were two deaths among the patients treated with lenvatinib, and there were five deaths among the patients treated with sorafenib; all deaths were determined to be due to disease progression and not drug-related. In both groups, all deaths were attributed to disease progression and were not considered to be related to treatment.

## Discussion

As there is a lack of real-world data on the use of TKIs, specifically lenvatinib, and sorafenib, for the treatment of DTC, which may preclude the use of these drugs in clinical practice, we conducted this retrospective chart review study to assess and compare the safety of both drugs in patients with DTC treated at a single third-level-of-care center in Korea.

In a real-world clinical setting, drugs are used according to comprehensive patient situations. Meanwhile, in clinical trials, strict compliance to protocol is prioritized. In routine clinical practice, physicians can manage AEs by adjusting the drug dosage, implementing treatment interruption periods, or adjusting the follow-up period once they take into consideration all the clinical aspects of each patient individually. Thus, the results of the present study are meaningful in that they provide insight into the safety profile of these TKIs in a real-world setting in patients with DTC who are not subject to strict eligibility criteria or strict clinical study conditions.

The AEs of any grade reported in patients treated with lenvatinib in the present study (diarrhea [82.6%], hypertension [78.3%], hand-foot skin reaction [56.5%], weight loss [52.2%], proteinuria [47.8%], and anorexia [43.5%]) were comparable with those reported in the SELECT trial (hypertension [67.8%], diarrhea [59.4%], fatigue or asthenia [59.0%], decreased appetite [50.2%], decreased weight [46.4%], and nausea [41.0%]) (12). Previous studies of patients treated with lenvatinib showed an increased risk of hypertension and proteinuria (17, 18).

The AEs reported in patients treated with sorafenib in the present study (hand-foot skin reaction [87.5%], diarrhea [62.5%], anorexia [60.4%], alopecia [56.3%], mucositis [52.1%], weight loss and generalized weakness [each, 50%], and hypertension [43.8%]) were comparable with those reported in the DECISION trial (hand-foot skin reaction [76.3%], diarrhea [68.6%], alopecia [67.1%], and rash or desquamation [50.2%]) (16). No new safety concerns were identified for either drug.

We observed some key differences in the safety profiles of lenvatinib and sorafenib. The incidence of hand-foot skin reaction, alopecia, and rash of any grade was significantly lower in patients treated with lenvatinib compared with those treated with sorafenib. The incidence of hypertension, QT prolongation, and proteinuria of any grade was significantly higher in patients treated with lenvatinib compared with those treated with sorafenib.

Dermatologic toxicities, such as rash, erythema, pruritus, acneiform rash, paronychia, telangiectasia, alopecia, changes in hair growth or pigmentation, skin discoloration, xerosis, and hand-foot skin reaction, particularly with TKIs targeting vascular endothelial growth factor receptor (VEGFR) or EGFR pathways, have been well-characterized (19–21). Hand-foot skin reaction and rash are symptomatic AEs that can affect the quality of life (QoL) of patients and hinder patient adherence to the treatment. A previous study on the impact of dermatologic AEs secondary to TKI treatment on QoL reported that dermatologic toxicities, including rash, xerosis, paronychia, and pruritus, adversely affected QoL. Among these AEs, rash was associated with the greatest decrease in QoL (22). Another study focusing on the clinical psychologist's perspective reported that dermatologic toxicities associated with TKIs had an impact on patients' physical, functional, emotional, and social well-being. Patients reported feeling worry, frustration, and depression because of their dermatologic symptoms (23). Such dose-dependent skin toxicities may lead to physical and psychosocial discomfort, which can result in dose reduction or treatment interruption (20).

Hypertension is another well-documented, characterized AE resulting from VEGFR inhibition (24). However, hypertension is generally asymptomatic and can be clinically managed with medication. A recently published exploratory analysis of the phase 3 lenvatinib SELECT trial data showed that although hypertension was indeed a clinically significant AE, treatment-related hypertension was significantly correlated with improved outcomes in patients with radioiodine-refractory DTC (24).

Dose reductions due to AEs were lower in patients treated with lenvatinib (34.8%) compared with sorafenib (56.3%). Thus, this difference in the safety profile of lenvatinib and sorafenib may have been the reason why the rate of dose reductions due to AEs was lower in patients treated with lenvatinib compared with sorafenib. Another plausible explanation for the lower dose reduction rate due to AEs in this patient group is that 100% of patients treated with lenvatinib initiated therapy at a dose of 20 mg.

All patients treated with lenvatinib (100%) received an initial dose of 20 mg (83% of the recommended initial dose). In contrast, 41.7% of the patients treated with sorafenib received an initial dose of 800 mg (100% of the recommended initial dose), 33.3% received an initial dose of 600 mg (75% of the recommended initial dose), and 25% received an initial dose of <400 mg (<50% of the recommended initial dose). The main reason most patients initiated lenvatinib treatment at a dose of 20 mg was economic burden. As of August 2017, the treatment costs of lenvatinib are being reimbursed by the National Health Institute of Korea (25). Furthermore, Kiyota et al. (28) conducted a subanalysis of Japanese patients enrolled in the global SELECT study, which revealed higher rates of certain adverse events such as hypertension and proteinuria, as well as a higher rate of dose reduction compared with non-Japanese patients. These results suggest that patients of east Asian descent may benefit from initiating lenvatinib at a lower dose, rather than undergoing subsequent dose reduction.

None of the patients treated with lenvatinib discontinued treatment because of disease progression. However, 35.4% of patients treated with sorafenib had to discontinue sorafenib treatment because of disease progression. The present results were comparable with those reported in two previous studies on lenvatinib in real-world clinical practice (26, 27).

## Study Limitations

This study had several limitations, including the limited generalizability of the results, problematic verification of information, difficulty establishing cause and effect, variation in the quality of information recorded by medical professionals, and other limitations inherent to the chart review design of the study. Further, in about half of the patients, lenvatinib was used as second-line treatment, while sorafenib was only used in naïve patients because of the market launch and reimbursement gap. This affected the number of patients in each group and may have also affected the safety results.

## Conclusions

AEs can occur frequently with lenvatinib and sorafenib; however, we observed that dermatologic AEs were significantly more frequent with sorafenib than lenvatinib. Similarly, hypertension was significantly more frequent with lenvatinib than sorafenib. Overall, safety outcomes were in line with previously reported clinical trial data, and most AEs were managed with dose modification and medical therapy. Most AEs of lenvatinib were manageable and observed within the first 6 months of treatment. Thus, it is important to monitor patients, particularly during the initial treatment period. In patients treated with lenvatinib, AEs such as hypertension and proteinuria warrant close monitoring and management if necessary.

## Ethics Statement

The study was carried out in accordance with the principles laid out in the World Medical Association's Declaration of Helsinki, Good Clinical Practice, and associated Korean regulations. This study was approved by the Institutional Review Board of Gangnam Severance Hospital (approval number 2017-0116-004). As data were obtained retrospectively, patient identities remained anonymous, and informed consent is not mandatory for retrospective studies in Korea, the institutional review board waived the need for informed consent.

## Author Contributions

SYK, S-MK, HC, H-SC, and CP contributed to the conception and design of the study. SYK, HC, B-WK, and YL organized the database. SYK and S-MK performed the statistical analysis, wrote the first draft of the manuscript, and wrote sections of the manuscript. All authors contributed to manuscript revision and read and approved the submitted version.

### Conflict of Interest Statement

The authors declare that the research was conducted in the absence of any commercial or financial relationships that could be construed as a potential conflict of interest.

## Footnotes

^1^Available online at: http://www.lenvima.com/pdfs/prescribing-information.pdf (accessed August 8, 2018)

^2^Available online at: https://www.accessdata.fda.gov/drugsatfda_docs/label/2013/021923s016lbl.pdf (accessed August 8, 2018)

## Abbreviations

AE: adverse event
DTC: differentiated thyroid cancer
ECOG: Eastern Cooperative Oncology Group
FTC: follicular thyroid cancer
PDGF: platelet-derived growth factor
PFS: progression-free survival
PTC: papillary thyroid cancer
QoL: quality of life
RAI: radioactive iodine
TKI: tyrosine kinase inhibitor
VEGF: vascular endothelial growth factor
VEGFR: vascular endothelial growth factor receptor.
